# 392. Guideline adaptation of fidaxomicin as first-line therapy for *Clostridioides difficile* infection in institutions across the United States

**DOI:** 10.1093/ofid/ofac492.470

**Published:** 2022-12-15

**Authors:** Angela Basir, Nicole Bradley, Yuman Lee

**Affiliations:** St. John's University, Queens, New York; St. John's University, Queens, New York; St. John's University, Queens, New York

## Abstract

**Background:**

In 2021, *Clostridioides difficile* infection (CDI) treatment guidelines were updated to recommend fidaxomicin over oral vancomycin as first-line therapy in initial and recurrent CDI episodes. Prior to this update, fidaxomicin use in management of CDI was limited, as cheaper alternatives were recommended as first-line therapy. The purpose of this study was to assess the guideline adaptation of fidaxomicin as first-line therapy in CDI management in institutions across the United States (US) and to identify barriers to its use.

**Methods:**

An electronic survey was distributed to members of the American Society of Health-System Pharmacists in April 2022. Baseline demographics, current practices, and barriers to fidaxomicin use were collected. Results were analyzed using descriptive statistics. Fisher’s Exact Test was used to compare categorical variables.

**Results:**

Based on 63 responses, majority of institutions (41/63, 65%) did not recommend use of fidaxomicin as first-line therapy for CDI. While most hospitals reported having institution specific CDI guidelines (48/63, 76%), over half of institutions with CDI guidelines still recommended oral vancomycin as first-line therapy (26/48, 54%). None of the institutions without CDI guidelines (0/15, 0%) recommended use of fidaxomicin as first-line therapy and majority of them (12/15, 80%) did not have fidaxomicin on formulary or restricted its use to ID services. Recommendation of fidaxomicin as first-line therapy was significantly higher in institutions with CDI guidelines versus those without (46% vs 0%, Fisher’s Exact 0.0006, p< 0.05).

Approximately 92% (58/63) of respondents reported at least 1 barrier to using fidaxomicin, with 39.6% (25/63) reporting 2 or more barriers to use. Barriers identified were cost (55/63, 87.3%), lack of formulary inclusion (14/63, 22.2%), lack of provider familiarity (13/63, 20.6%) and inability of patients to afford upon discharge (7/63, 11.1%).

**Conclusion:**

Majority of institutions do not currently recommend use of fidaxomicin as first-line therapy for CDI despite the recent guideline updates. Institutions with CDI treatment guidelines were more likely to recommend use fidaxomicin as first-line therapy. Cost and lack of formulary inclusion were cited as the most frequent barriers to fidaxomicin use.

**Disclosures:**

**All Authors**: No reported disclosures.

